# *Notes from the Field:* Contact Investigation for an Infant with Congenital Tuberculosis Infection — North Carolina, 2016

**DOI:** 10.15585/mmwr.mm6723a5

**Published:** 2018-06-15

**Authors:** Jessica L. Rinsky, Darlene Farmer, Jessica Dixon, Jean-Marie Maillard, Thomas Young, Jason Stout, Amina Ahmed, Aaron Fleischauer, Jennifer MacFarquhar, Zack Moore

**Affiliations:** ^1^Epidemic Intelligence Service, CDC; ^2^Division of Public Health, North Carolina Department of Health and Human Services; ^3^Wake County Human Services, Raleigh, North Carolina; ^4^WakeMed Health & Hospitals, Raleigh, North Carolina; ^5^Department of Medicine, Duke University School of Medicine, Durham, North Carolina; ^6^Carolinas HealthCare System, Charlotte, North Carolina; ^7^Office of Public Health Preparedness and Response, CDC.

In November 2016, hospital A notified the North Carolina Division of Public Health (NCDPH) that annual tuberculosis screening of neonatal intensive care unit (NICU) staff members identified six health care staff members with newly positive tuberculin skin tests (TSTs). All six staff members had cared for an infant in whom a diagnosis of congenital tuberculosis was made after death. NCDPH worked with county health departments and hospital A to conduct a contact investigation.

The infant was born at hospital A in July 2016 at 25 weeks’ gestational age to a mother originally from a country with high tuberculosis prevalence. After delivery, the mother developed respiratory distress that required intubation; a bronchoalveolar lavage (BAL) specimen was negative for acid-fast bacilli. The infant was admitted to the NICU with fever and respiratory failure, supported by high-frequency oscillatory ventilation, and died after 17 days. One month after delivery, *Mycobacterium tuberculosis* was isolated from a culture of the mother’s BAL specimen. A contact investigation around the mother at the time of diagnosis identified no positive TST test results among health care staff members. Microscopic examination of the stored placenta revealed acid-fast bacilli. During investigation, medical records obtained from fertility treatment 2 years earlier at a hospital in another state indicated that the mother had granulomatous salpingitis on histopathology, consistent with genitourinary tuberculosis; delivery and NICU staff members were unaware of the mother’s medical history. No contact investigation had been performed around the infant before this investigation.

A contact was defined as a person who treated or spent time in the open NICU with the index infant. Health care staff members and volunteers identified as contacts were screened with TSTs; tests with induration ≥5 mm was considered a positive result. Persons with a history of positive TST results were screened for tuberculosis symptoms by clinical examination. For NICU patients identified as contacts, NCDPH recommended a TST and interferon-gamma release assay (IGRA), clinical evaluation including a chest radiograph, preemptive treatment with 9 months of isoniazid, and clinical monitoring until age 2 years. Preemptive treatment was recommended because of concerns about false negative results among infants, who are at increased risk for developing active tuberculosis (*1*–*4*). NICU visitors identified as contacts were evaluated with IGRAs.

In total, 132 of 135 (98%) health care staff members were evaluated; seven (5%), including the original six NICU staff members identified through annual tuberculosis screening, had a newly positive TST result (induration range = 10–20 mm), and all had performed at least one aerosol-generating procedure (e.g., intubation or open suctioning) on the index infant. None of the staff members with positive TSTs had been exposed to the mother or reported other known exposures. All 29 NICU volunteers were notified of their exposure; 15 (52%) were screened for tuberculosis infection at hospital A, and all were negative.

Among 23 NICU visitors tested, one (4%) had a positive IGRA. This visitor reported no other risk factors for tuberculosis infection and had spent multiple hours per day during 11 days sitting with an infant adjacent to the index infant. All adults who tested positive for tuberculosis infection received latent tuberculosis treatment through local health departments.

Twenty-six infants were present in the NICU during the index infant’s hospitalization. Families of 25 infants (96%) were notified; one family could not be located. Clinical assessment was performed on 22 (85%) infants, including 16 who received a TST and IGRA, three who received only IGRA, and three who received only TST. None had a positive screening test or evidence of active disease. Eighteen (82%) of the 22 infants began preemptive latent tuberculosis treatment, and four (18%) entered clinical monitoring without treatment.

Annual TST screening of health care staff members prompted an investigation that revealed likely transmission of tuberculosis from an infant with congenital infection to seven NICU staff members and one visitor. Factors that might have contributed to this transmission event include congenital infection, which is associated with high bacterial load, multiple aerosol-generating procedures, and respiratory support using a high-frequency oscillatory ventilator with unfiltered exhaust (*5*).

Congenital tuberculosis is rare (*1*,*6*); however, transmission from infants with congenital infection to health care workers has been documented (*1*,*5*,*7*). Transmission to visitors or other patients has not previously been documented except by exposure to contaminated medical devices (*1*,*2*,*5*). Patients and visitors were considered contacts here because of evidence of transmission to multiple health care staff members and aerosol-generating procedures performed in the NICU.

Tuberculosis has been associated with infertility, particularly in high-prevalence countries. Early detection and treatment of latent and active tuberculosis infections among pregnant women and those seeking to become pregnant can prevent transmission to their infants. Medical providers should also consider a thorough evaluation for tuberculosis among infants born to mothers who have epidemiologic risk factors for tuberculosis and a compatible clinical presentation. Even if tuberculosis is not suspected, routine use of control measures (e.g., closed suctioning and filtering air exhaust ports from ventilators) might be considered to reduce the potential for exposure. Finally, when exposure cannot be prevented, adherence to contact investigation guidelines is important (*8*) to rapidly identify and evaluate contacts, including visitors who shared airspace with an infant with congenital tuberculosis infection during a prolonged period or during aerosol-generating procedures.
